# Cadmium, von Willebrand factor and vascular aging

**DOI:** 10.1038/s41514-023-00107-3

**Published:** 2023-06-01

**Authors:** Xia Wang, Maria N. Starodubtseva, Carolyn M. Kapron, Ju Liu

**Affiliations:** 1 Institute of Microvascular Medicine, The First Affiliated Hospital of Shandong First Medical University& Shandong Provincial Qianfoshan Hospital, Jinan, China; 2 Gomel State Medical University, Gomel, Belarus; 3 Institute of Radiobiology of NAS of Belarus, Gomel, Belarus; 4 Department of Biology, Trent University, Peterborough, ON Canada

**Keywords:** Ageing, Risk factors, Ageing

## Abstract

Vascular aging is a major contributing factor to cardiovascular disease. The aged blood vessels, characterized by vascular wall thickening and stiffening, are instigated by endothelial cell dysfunction induced by oxidative stress and inflammation. von Willebrand Factor (vWF) is a glycoprotein known for its role in coagulation, and plasma levels of vWF are increased with age. Elevated vWF promotes thrombosis, atherosclerotic plaque formation, inflammation and proliferation of vascular smooth muscle cells. Cadmium (Cd) is an environmental pollutant associated with increased morbidity and mortality of cardiovascular disease. At low concentrations, Cd activates pro-survival signaling in endothelial cells, however enhances intima-media thickness and atherogenesis. A non-cytotoxic dose of Cd also increases endothelial vWF expression and secretion in vivo and in vitro. In this review, we summarize the molecular mechanisms underlying vWF-promoted vascular aging-associated pathologies and Cd-induced vWF expression. In addition, we propose that exposure to low-dose Cd is a risk factor for vascular aging, through elevation of plasma vWF.

## Vascular aging

Vascular aging, the decline in vascular structure and function with age, contributes to cardiac and peripheral vascular diseases^1^. In aging vessels, increased intima-media thickness (IMT) occurs linearly (~5 μm year^–1^) with older age, accompanied by vascular smooth muscle cell (VSMC) proliferation, and resultant increase of subendothelial extracellular matrix (ECM)^2,3^. The aged vessels also exhibit elastin fragmentation and calcification, leading to increased vessel stiffness and reduced compliance^4^. Age-related mechanical and adhesive properties of vascular cells also increases vascular stiffness, which destabilizes endothelium and subsequently promotes leukocyte transmigration and atherosclerosis^5,6^.

Vascular endothelial cell (EC) dysfunction is an early manifestation of vascular aging that precedes the structural remodeling and stiffness^4^. In the elderly, EC injury leads to malfunction of vascular tone, vascular permeability, and hemostasis. Nitric oxide (NO) is a major vasodilator released by ECs, and decrease of NO production impairs endothelium-dependent dilation (EDD) in vascular aging^7^. Age-dependent blood-brain barrier breakdown occurs initially in the hippocampus, a region critical for learning and memory^8^. In addition, endothelium-derived anticoagulant proteins including prostacyclin and thrombomodulin are down-regulated in vascular aging^9^. Whereas the expression of procoagulants such as von Willebrand Factor (vWF), thromboxane A2 and plasminogen activator inhibitor-1 are increased, favoring the development of thrombosis in vascular aging^10–12^.

Oxidative stress and inflammation are strongly associated with age-related endothelial dysfunction and vascular stiffening^13^. Aging is accompanied with increased production of reactive oxygen species (ROS) by reduced nicotinamide adenine dinucleotide phosphate (NADPH) oxidases and mitochondria, which reduces NO bioavailability and impairs EDD^14^. Oxidative stress also activates proinflammatory signaling pathways, including nuclear factor-κB (NF-κB), and increases proinflammatory cytokine gene expression including tumor necrosis factor-α (TNF-α), interleukin-1 (IL-1) and interleukin-6^15,16^. Both inflammatory cytokines and ROS induce the secretion of matrix metalloproteinase and transforming growth factor β1 that mediate ECM remodeling including elastin fragmentation, collagen accumulation and calcification^17^. Increased NF-κB activity upregulates the expression of endothelial adhesion molecules to facilitate leukocyte adhesion and transmigration^15^. Immune cells infiltration exacerbates the presence of inflammatory cytokines and the production of ROS, thus creating a vicious feed-forward cycle to accelerate vascular aging^13^. In addition, genomic instability, epigenetic alterations, deregulated nutrient sensing, stem cell dysfunction, and autophagy may involve in the development of vascular aging, and have been summarized in recent reviews^18,19^.

Cadmium (Cd) is a well-known hazardous pollutant^20^. The vascular endothelium is one of the major targets of Cd^21^. Epidemiological studies suggest adverse effects of environmental Cd exposure on age-related cardiovascular diseases in the general population, raising concerns about the validity of the current safe intake level^22–25^. Our group and others demonstrated that low-dose Cd upregulates endothelial expression of vWF, a key contributor of vascular aging-associated pathologies^26,27^. Therefore, we propose that environmental Cd exposure is a risk factor for vascular aging, possibly by elevation of vWF expression.

## vWF

vWF is a multimeric glycoprotein expressed in ECs and megakaryocytes^28^. ECs are the primary source of plasma and subendothelial vWF. Following synthesis in ECs, vWF is either constitutively secreted or stored in Weibel-Palade bodies (WPBs), from where it is released into plasma and basement membrane upon activation^28^. Increased plasma vWF is a hallmark of EC dysfunction. Plasma vWF regulates hemostasis by facilitating platelet adhesion and coagulation factor VIII (FVIII) stabilization^29,30^. Subendothelial vWF mediates initial attachment of platelet to the basement membrane after EC injury^31^. Deficiency of vWF, either quantitative or qualitative, causes a bleeding disorder known as von Willebrand disease (VWD)^32^.

Plasma vWF level and vWF expression in ECs is increased with age. Plasma vWF antigen shows a gradual increase of 1-2% per year in healthy human, and is accompanied by enhanced functional activity^10,33,34^. A substantial increase in vWF levels is found in both men and women after midlife (above 40 years of age)^33^. Increased plasma vWF levels have also been observed in patients with type 1 VWD where vWF level is normalized with advancing age, but not in type 2 and 3 VWD^35^. Notably, immunohistochemical analysis of cellular vWF expression demonstrated significantly elevated staining intensity of vWF in lung vasculature in adults compared to that in children^36^. In addition, age-related increase in vWF expression in brain ECs and in hepatic sinusoidal ECs have been documented in mice, rats, human, and non-human primates^37–39^.

## The roles of vWF in vascular aging-associated vascular pathologies

vWF is involved in several age-related vascular pathologies. Plasma levels of vWF are positively correlated with IMT and arterial stiffness in human cohorts^40,41^. In addition, higher vWF levels are associated with artery calcification and ischemic stroke^42^. Antibodies against vWF improve EC functions in animal models of vascular injury and patients with stable angina^43,44^.

vWF alters its conformation in response to hydrodynamic forces in the bloodstream^45,46^. The shear stress is proportional to blood flow velocity and viscosity, and inversely proportional to the blood vessel diameter^47^. Variations in shear stress and hydrodynamic forces occur near arterial bifurcations, branch mouths, and curvatures, which are associated with age-associated vascular remodeling^47^. Under elevated shear stress flow conditions, the structure of vWF is altered from a globular form (collapsed conformation) to a stretched linear conformation^46^. This structural transition correlates with the increased vWF adhesiveness to collagen and platelets^45^. A pathological increase in the adhesiveness of vWF to platelets in the blood circulation leads to thrombosis, thrombotic thrombocytopenia, and organ failure^45,48^.

## vWF promotes thrombosis

Vascular aging increases the risk to develop venous and arterial thrombosis in the older population^1,49^. Age-associated production of vWF may cause increased thrombogenicity^50^. After detachment of aged ECs from the vascular wall, subendothelial vWF is exposed to the bloodstream, and directly initiates platelet adhesion to the subendothelial tissue by interaction with platelet glycoprotein (GP) Ib^31^. Plasma vWF also binds to the subendothelial collagen and facilitates platelet adhesion^51^. The vWF-mediated low-affinity adhesion induces the activation of platelet αIIbβ3 integrin that in turn binds to fibrinogen, fibrin and vWF to stabilize platelet adhesion^29^. vWF immobilized on adherent activated platelets provides attachment sites for additional circulating platelets, facilitating platelet aggregation and thrombus formation^29^. Under high shear rates, platelet adhesion and aggregation are more dependent on vWF^52^. At shear rates over 1,0000/s, a condition that occurs only in stenotic arteries, thrombus formation is exclusively mediated by vWF-GPIb interaction^52^. In addition, plasma FVIII levels increase with age, and this increase is dependent on vWF levels^34^. In circulation, FVIII forms a complex with vWF, which protects FVIII from proteolytic degradation^30^. Upon vascular injury, activated FVIII (FVIIIa) dissociates from vWF, and binds to FIXa on the phospholipid membrane of activated platelets to form the factor Xase complex^53^. FVIIIa within the factor Xase complex stabilizes the FIXa active site, and serves as a molecular bridge between FIXa and its substrate FX, facilitating FXa generation^53^. FXa in complex with FVa activates prothrombin to thrombin^54^. Thrombin cleaves fibrinogen and yields monomeric fibrin, which deposits and polymerizes to stabilize the platelet-rich thrombus^54^. vWF also binds to the apical surface of activated ECs, where it mediates platelet adhesion on intact endothelial surface and facilitates thrombus formation in the absence of EC injury^55^. Thus, increased vWF in the microenvironment of aged ECs may promote platelets aggregation and subsequent thrombus formation.

## vWF modulates vascular remodeling

In aged blood vessels, VSMCs switch from a quiescent contractile phenotype to a synthetic phenotype characterized by increased cell proliferation, migration, and production of ECM proteins, promoting intimal thickening^3^. vWF is constitutively released into subendothelial space^28^, and plasma vWF penetrates into the intima of vessel walls through intercellular gaps caused by ECs injury^56,57^. Age-related accumulation of vWF is observed in porcine aortic valve subendothelium, and associated with valvular interstitial cell calcification^58^. In ligation-induced carotid intimal hyperplasia, vWF expression in ECs and vWF deposition in neointimal ECM is significantly elevated^56^. The level of vWF is positively correlated with the degree of intimal hyperplasia^56^. Increased expression and deposition of vWF in hyperplastic intima is also noted in atherosclerotic plaques, vascular grafts, and balloon angioplasty^57,59–61^. vWF deficiency in mice leads to a decreased outward remodeling and VSMC proliferation^62^. In vitro studies show that vWF directly increases VSMC proliferation and migration with a dose-response effect^56,63^. vWF interacts with integrin αvβ3 on VSMC to facilitate VSMC adhesion to the endothelial basement membrane^64^. In addition, vWF binds to the LRP4-receptor on VSMC, which in turn triggers integrin αvβ3 signaling to promote VSMC proliferation^63^. Blockage of αvβ3 signaling inhibits the adhesion and proliferation of VSMC induced by vWF^63,64^.

## vWF facilitates vascular inflammation

Chronic, low-grade inflammation in the vascular wall is a hallmark of vascular aging^13^. As a critical early step in chronic vascular inflammation, leukocyte-endothelial interaction is increased in aging vessels due to enhanced endothelial adhesiveness^65^. The leukocyte subsequently releases cytokines, proteases and ROS, which induce vascular aging^39,65^. vWF promotes leukocyte-endothelial interaction via multiple mechanisms. vWF directs biogenesis of WPBs that contain P-selectin, a known inflammation mediator^66^. vWF deficiency impairs P-selectin translocation to EC surface and reduces leukocyte recruitment in early phases of inflammation^67^. In addition, endothelium-associated vWF creates an adhesive surface for neutrophils and mediates their rolling through P-selectin glycoprotein ligand (PSGL)-1 and stable adhesion through β2-integrins under static and low-shear conditions^68^. With a high shear stress in arteries and arterioles, endothelium-associated vWF captures platelets which promote neutrophil rolling via P-selectin/PSGL-1 and neutrophil adhesion via GPIb/Macrophage (MAC)-1 interactions^69^. Moreover, vWF promotes neutrophil extravasation in a strictly platelet and GPIb dependent way^70^. Subendothelial vWF inhibits tight junction protein claudin-5 expression thus destabilizes endothelial barrier^71^. vWF also promotes alternative complement pathway activation and stabilizes neutrophil extracellular traps (NETs), both conditions effect age-related vascular inflammation^72–74^. Notably, anti-vWF treatment showed a vascular anti-inflammatory effect both in prophylactic and therapeutic administration^75^.

## vWF enhances atherosclerosis

Aging is an independent risk factor for the development of atherosclerosis^1^, and age-associated vascular wall degeneration is also aggravated by the presence of atherosclerosis^76^. In population-based stuides, plasma levels of vWF are markedly higher in patients with atherosclerosis, and vWF are positively correlated with the plaque thickness and stenosis area^77,78^. In carotid and coronary arteries from human, large numbers of WPBs are present in ECs at sites of atherosclerotic lesions^79^. Increased vWF staining is also observed in the intima of human atherosclerotic arteries^63,80^. Although clinical reports are inconsistent on whether VWD patients are protected from atherosclerosis^81–84^, pigs with severe VWD show a decreased number of aortic plaques on a cholesterol-rich diet and mice deficient of vWF display reduced fatty streaks^85,86^. Endothelial vWF is up-regulated in response to hypercholesterolemia before the advent of fatty streaks and recruits circulating platelets to the endothelium at atherosclerosis-prone sites^87^. vWF-bound platelets interact with monocytic PSGL-1 via P-selectin and with monocytic MAC-1 via GPIb, thereby promoting monocytes attachment on endothelium and transmigration^69^. Recruited monocytes in subendothelium differentiate into macrophages to engulf oxidized low-density lipoprotein (oxLDL) by scavenger receptor, which gradually leads to accumulation of LDL-derived cholesterol and subsequent lipid-rich core formation^76^. vWF deficiency reduces the presence of monocytes in the fatty streaks^86^. Hence, vWF may facilitate monocytes transmigration to support the development of atherosclerotic lesions.

vWF is a multitasker in cellular processes, and the resulting pathophysiological consequences of vWF dysregulation are still controversial. Further investigation is needed to clarify the detailed roles and the underlying mechanisms of vWF in vascular aging-associated pathologies.

## Low-dose Cd induces endothelial vWF expression and secretion

A positive correlation between cigarette smoking, a primary source of Cd exposure, and plasma vWF levels has been detected^88^. Low-dose Cd (<10 μM) does not induce ECs death, but increases VEGF receptor-2 expression and promotes angiogenesis^89^. The effect of low-dose Cd exposure on endothelial vWF expression and secretion was examined^26^. Experimental mice were fed with cadmium chloride (CdCl_2_) in distilled water and displayed elevated expression of vWF in the endothelium of lung and kidney. In vitro, CdCl_2_ at concentrations as low as 1 μM (the rough concentration found in the arterial intima of smokers were 1.5 μM^90^) induces the expression and secretion of vWF in human umbilical vein endothelial cells (HUVECs). Mechanistically, CdCl_2_ exposure increases ETS-related gene (ERG) expression and enhances its binding to the -56 ETS-motif on the promoter of the *vWF* gene (*vWF*), and thereby elevates *vWF* transcription^26^. ERG, a member of the ETS family of transcription factors, is specifically expressed in ECs^91^. The cis-acting element GGAA/T, situated at -56 in the core promoter of *vWF*, is bound and activated by ERG^92^. Interference of ERG expression reduces vWF expression and this effect is abolished by mutations of the -56 ETS-motif of the *vWF* promoter^92^. Although ERG is known for its role in endothelial homeostasis, aberrant expression of endothelial ERG has been observed in pathological conditions including vascular malignancies^93–97^, arterial calcification^98^, and tumour neovascularization^99,100^. In addition, overexpression of ERG in the Xenopus embryo results in developmental defects and ectopic endothelial differentiation^101^.

The human *vWF* promoter contains multiple cis-regulatory sequences that positively or negatively regulate gene expression. A GATA transcription factor binding site, situated at position +220 in the first exon of *vWF*, mediates *vWF* transcription in an ECs-specific manner^102^. Transcription factor NF-κB interacts with the -1793 sequence to repress vWF expression^103^. In contrast to ERG, the levels of GATA3 and NF-κB are not effected by low-dose Cd^26^. Nevertheless, other mechanisms may also mediate low-dose Cd induced vWF expression, including nuclear factor 1-like protein, histone H1-like protein and nuclear factor-Y transcription factor, which bind to the core promoter of *vWF*^104–106^.

## Environmental Cd exposure is a risk factor for vascular aging

Cd is a toxic metal that occurs naturally in sulfide ores^107^. Since the 1940s, Cd has been widely utilized in production of batteries, alloys, coatings, plating, and plastic stabilizers^20^. In addition, large amount of Cd is released into environment through vehicle exhaust, pesticides and fertilizers^20^. Currently, Cd is ranked the seventh in environmental toxic pollutants^108^. For population without occupational exposure, environmental Cd enters human body primarily through ingestion of Cd-contaminated food and water^20^. Environmental Cd exposure is particularly high in East Asian due to the dietary exposure via consumption of Cd-contaminated rice, fish, and shellfish^109^. Cigarette smoking is the dominant source of inhaled Cd in addition to ambient air pollution^20^. After absorption, Cd is distributed throughout the body via bloodstream, progressively accumulating mainly in the kidney and liver^110^.

Cd is transported in blood plasma bound to proteins or as free ions^21^. The U.S. OSHA safety standard is currently 5 μg/L (44.5 nmol/L) for blood Cd (bCd)^111^. In the general US population, bCd at even low levels are associated with increased prevalence of peripheral artery disease (PAD)^23^. Tellez-Plaza et al. reported that the prevalence of PAD increases with bCd (within 1 μg/L) in a dose-dependent manner^25^. In a 5-year follow-up of 64-year-old women from Sweden, prevalence of PAD is significantly higher in the group with high bCd (0.44 to 4.07 μg/L) than that with low bCd (0.08 to 0.25 μg/L)^24^. In addition, low-level Cd exposure of general populations is associated with myocardial infarction, stroke, heart failure, and cardiovascular mortality^112–114^. In healthy young female subjects with an average age of 20.6 years, plasma levels of Cd are positively correlated to IMT^115^. Environmental Cd exposure is also associated with atherosclerotic plaques development in middle-aged men and women from Sweden^22^. Cd-induced atherosclerotic changes have been reported in the coronary arteries of rabbits, and the aorta of Wistar rats and White Carneau pigeons^116–119^. In addition, Cd-fed *ApoE*^*−/−*^ mice exhibit significantly increased area of aortic plaques^115,120,121^. These evidences suggest that, Cd exposure, even at relatively low levels, may increase vascular aging-associated pathologies.

**Fig. 1:**
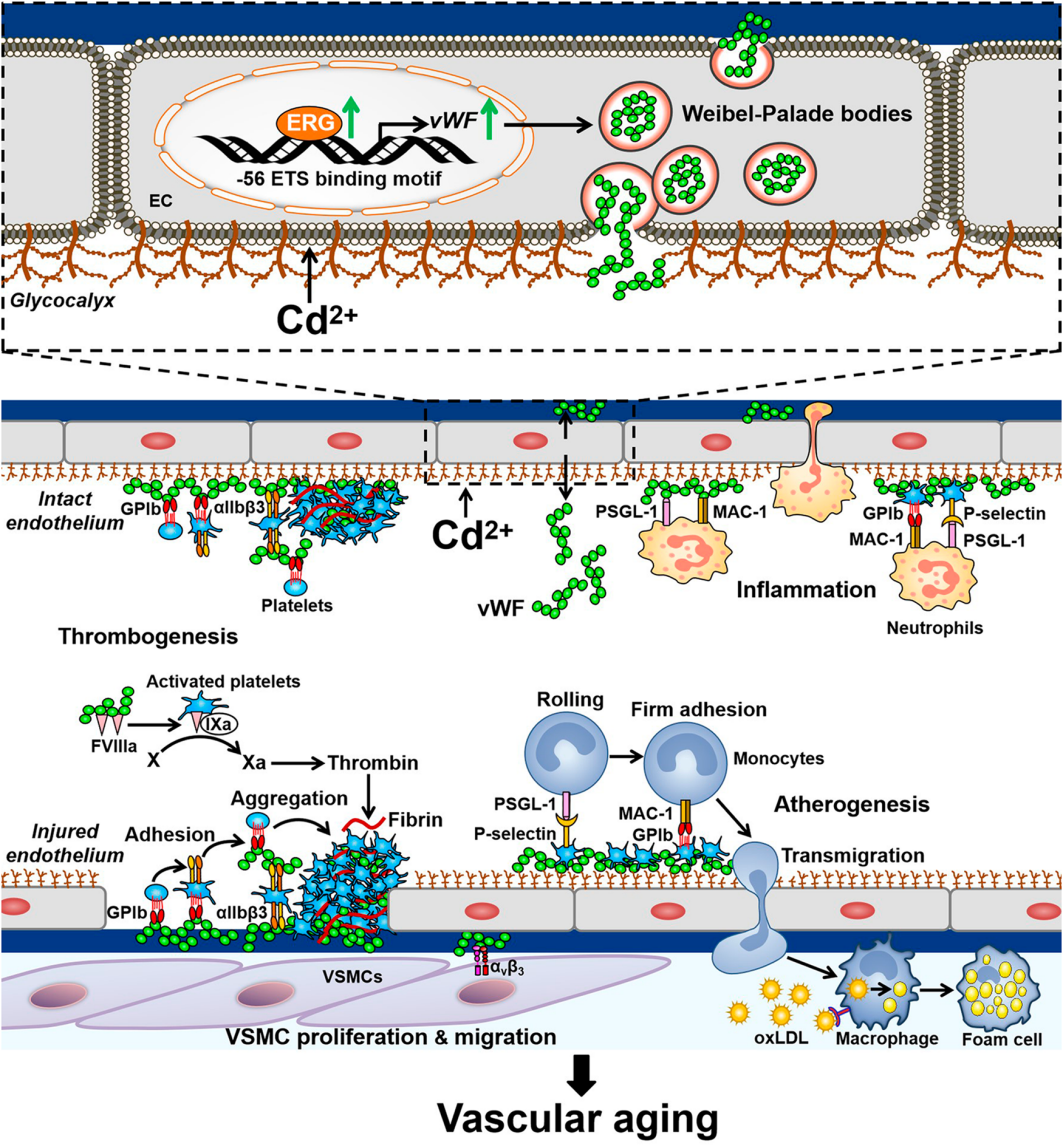
Low-dose cadmium (Cd) contributes to vascular aging through up-regulation of vWF. Low-dose Cd exposure induces the expression of ETS-related gene (ERG), which transcriptionally active von Willebrand Factor (vWF) expression and secretion. At sites of endothelial injury, plasma and subendothelial vWF interacts with circulating platelets via GP Ib and integrin αIIbβ3 to promote platelet adhesion and aggregation. Plasma vWF also binds to intact but activated endothelium, where it facilitates platelets binding under flow and intravascular thombus formation. vWF bound platelets are activated to assist the rolling and attachment of monocytes to the endothelium via P-selectin, and to promote monocyte extravasation via GPIb binding. Recruited monocytes differentiate into macrophages to engulf oxidized low-density lipoprotein (oxLDL), leading to the accumulation of LDL-derived cholesterol and lipid-rich core formation. vWF also mediates neutrophil attachment and emigration through platelet activation or direct interaction with neutrophils via P-selectin glycoprotein ligand (PSGL)-1 and Macrophage (MAC)-1, resulting in a proinflammatory state of the endothelium. In addition, subendothelial vWF promotes vascular smooth muscle cells (VSMCs) proliferation and migration in an αvβ3-dependent manner, leading to increased intima-media thickness and stiffness.

The vascular endothelium lines the lumenal surface of all blood vessels and form the capillary networks that deliver oxygen and nutrients to tissues of the body^4^. As a toxicant circulating in blood, Cd interacts directly with vascular ECs, thus targeting the vascular system^21^. Acute exposure to high-level Cd causes ECs apoptosis or necrosis and results in hemorrhage in various tissues^21^. Whereas at non-cytotoxic concentrations, Cd activates pro-survival signaling in ECs, leading to enhanced cell proliferation^89^. Low-dose Cd exposure does not induce toxicity of ECs, but significantly increases endothelial vWF expression and secretion^26^. In addition, Cd treatment increases the number and exocytosis of WPBs, the storage receptacle of vWF, in ECs of thoracic aorta of rats^27^. Therefore, environmental Cd exposure increases vWF expression, and may subsequently accelerate vascular aging (Fig. 1). However, the role of Cd exposure on vWF-dependent vascular pathologies, e.g., platelet adhesion, shall be validated in the future studies.

## Discussion

Vascular aging is associated with inflammation and oxidative stress, both of which increase vWF secretion^13^. Although proinflammatory cytokines such as TNF-α and IL-1 induces oxidative stress and stimulates vWF secretion by the stimulation of exocytosis of WPBs, they potently inhibit the expression of vWF, leading to a transient elevation but long-term down-regulation of plasma vWF^122,123^. NF-κB signaling mediates oxidative stress and is activated by endogenous hydrogen peroxide (H_2_O_2_) during aging^15^, but activation of NF-κB signaling also represses vWF expression^103^. Although a high concentration of exogenous H_2_O_2_ (≥ 200 μM) increases endothelial vWF expression, it induces substantial cell death in ECs and is unlikely to be reached in human pathophysiology^124^. Cd at a concentration lower than 10 μM does not induce cell death but activates pro-survival signaling and increases proliferation of ECs^89^. Low-dose Cd-induced *vWF* transcription may constantly produce additional vWF protein to sustain age-associated increase of vWF levels.

Plasma vWF levels are increased with aging. Elevated vWF induces vascular aging-associated pathologies by promotion of thrombus formation, vascular remodeling, vascular inflammation and atherogenesis. Cd is a widespread environmental pollutant, and associated with age-related vascular diseases. At non-cytotoxic concentrations, Cd induces the expression and secretion of vWF in ECs. Therefore, environmental Cd exposure may accelerate vascular aging through the elevation of endothelial vWF. Reduction of Cd exposure and therapeutic approaches to decrease vWF expression may decelerate vascular aging.

## Data Availability

All data here disclosed are published in the literature as indicated in the references section.
